# Synthetic Fructoborate Administration: Effects on Cardiac Remodeling and Extracardiac Responses in Isoproterenol-Induced Cardiac Hypertrophy

**DOI:** 10.3390/ph19091437

**Published:** 2026-09-10

**Authors:** Clara Ortega-Camarillo, Guadalupe Díaz-Rosas, Beatriz Muñiz-Reyes, Perla L. Nieto-Lara, R. Ivan Cordova-Chavez, Andrei Bită, Marvin A. Soriano-Ursúa, Renata Saucedo, Alejandro Ávalos-Rodríguez, José A. Morales-Serna, Ricardo Chávez-García, Alejandra Contreras-Ramos

**Affiliations:** 1 Unidad de Investigación Médica en Bioquímica, Hospital de Especialidades, Centro Médico Nacional Siglo XXI, Instituto Mexicano del Seguro Social, Ciudad de Mexico 06720, Mexico; clara.ortegac@imss.gob.mx; 2 Laboratorio de Investigación en Biología Molecular, Hospital Infantil de México Federico Gómez, Ciudad de Mexico 06720, Mexico; gudiro2@yahoo.com.mx (G.D.-R.); betymuniz4@gmail.com (B.M.-R.); perla.nieto@cua.uam.mx (P.L.N.-L.); joseantonio.moralesserna@gmail.com (J.A.M.-S.); 3 División de CBS, Universidad Autónoma Metropolitana-Iztapalapa, Ciudad de Mexico 09310, Mexico; 4 División de CNI, Universidad Autónoma Metropolitana-Cuajimalpa, Ciudad de Mexico 05348, Mexico; 5 Sección de Estudios de Posgrado e Investigación, Escuela Superior de Medicina, Instituto Politécnico Nacional, Plan de San Luis y Díaz Mirón s/n, Ciudad de Mexico 11340, Mexico; ivancoch@hotmail.com (R.I.C.-C.); msoriano@ipn.mx (M.A.S.-U.); 6 Faculty of Pharmacy, University of Medicine and Pharmacy of Craiova, 2 Petru Rareş Street, 200349 Craiova, Romania; andrei.bita@umfcv.ro; 7 Unidad de Investigación Médica en Enfermedades Endocrinas, Hospital de Especialidades, Centro Médico Nacional Siglo XXI, Instituto Mexicano del Seguro Social, Ciudad de Mexico 06720, Mexico; renata.saucedo@imss.gob.mx; 8 Departamento de Producción Agrícola y Animal, División de Ciencias Biológicas y de la Salud, Universidad Autónoma Metropolitana-Xochimilco, Ciudad de Mexico 04960, Mexico; avalosr@correo.xoc.uam.mx (A.Á.-R.); rchavezg@correo.xoc.uam.mx (R.C.-G.)

**Keywords:** synthetic fructoborate, boron, cardiac remodeling, left ventricular hypertrophy, isoproterenol, oxidative stress, extracardiac responses

## Abstract

**Background:** Synthetic fructoborate (sFB) is a boron-containing compound with reported biological effects, but its influence on cardiac remodeling remains poorly characterized. **Objective:** To evaluate the cardiac effects of short-term sFB administration and compare cardiac and extracardiac responses when sFB was administered before or after isoproterenol-induced cardiac hypertrophy. **Methods:** Male BALB/c mice received sFB (17, 34, or 68 mg/kg) for 3 days to assess cardiac morphology, p38 MAPK phosphorylation, Atrial *Natriuretic Peptide (ANP)* and *Natriuretic Peptide B (BNP)* mRNA expression, and oxidative stress-related markers. In a second experiment, cardiac hypertrophy was induced with isoproterenol (50 mg/kg, intraperitoneally, for 7 days), and sFB (34 mg/kg for 3 days) was administered before or after hypertrophy induction. Cardiac and extracardiac histological, molecular, and biochemical responses were evaluated. **Results:** Short-term sFB administration did not produce a consistent pattern of cardiac injury. The p-p38 MAPK/p38 MAPK ratio decreased, and 17 mg/kg sFB increased plasma glutathione (GSH) levels. When administered after hypertrophy induction, sFB reduced left ventricular wall thickness, cardiomyocyte area, and collagen I and III deposition and increased ventricular lumen area. Administration before isoproterenol exposure produced a different response, with some preserved structural parameters but persistent collagen deposition and increased expression of remodeling-associated genes. Extracardiac responses were heterogeneous and tissue-dependent, with notable hepatic alterations under some conditions. **Conclusions:** The effects of sFB on isoproterenol-induced cardiac hypertrophy depended on the timing of administration, with more consistent attenuation of structural remodeling when administered after hypertrophy induction. Further studies are required to establish its long-term safety, pharmacokinetics, and potential therapeutic relevance.

## 1. Introduction

Boron (B) is a naturally occurring element found in biological systems and is obtained primarily through diet and drinking water [1,2]. A broad range of natural and synthetic boron-containing compounds has been investigated for biological and pharmacological applications. Depending on their chemical structure, these compounds have shown antimicrobial, antitumor, antiparasitic, anti-inflammatory, and other biological activities, including effects on cardiovascular and metabolic processes [3,4,5,6].

**Figure 1:**
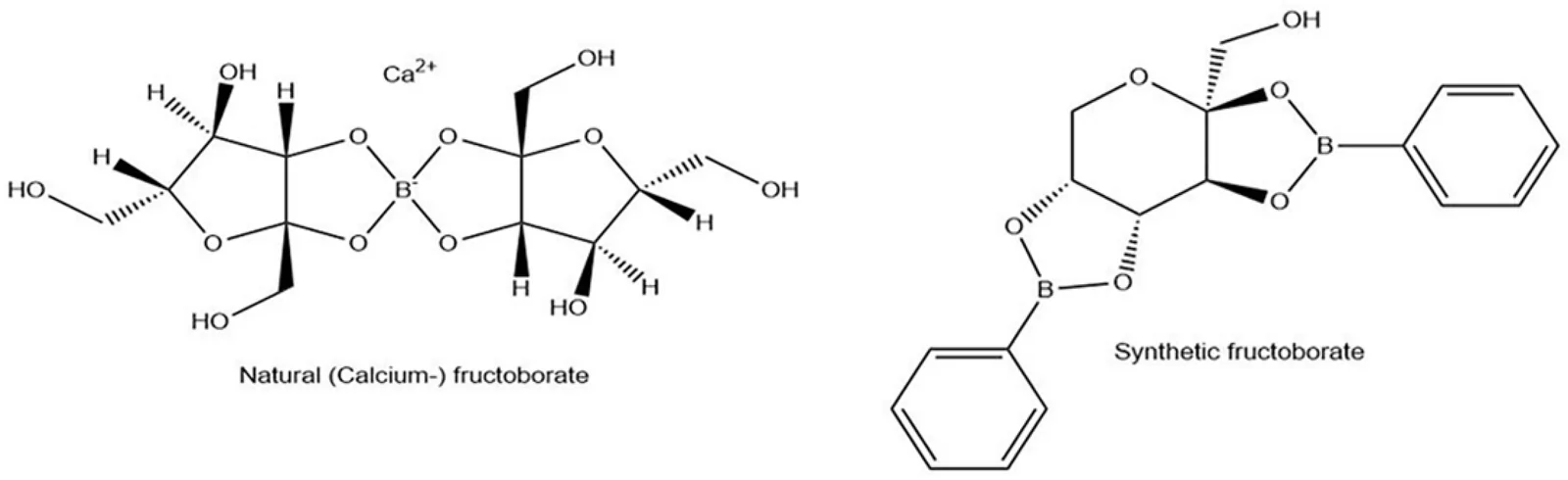
Chemical structures of natural and synthetic fructoborates.

The biological properties of boron-containing compounds are strongly influenced by their chemical structure and by the ability of boron-containing functional groups to interact with biological targets [7,8]. These characteristics have supported the development of several boron-containing compounds for biomedical applications [8,9]. In this context, Córdoba-Chávez et al. [10] synthesized a fructose-derived boron-containing compound (FB-1), hereafter referred to as synthetic fructoborate (sFB), containing two boronate moieties associated with a fructose-derived scaffold (Figure 1). Previous studies have provided initial information on the acute toxicity and biological effects of sFB [10], including effects associated with ion homeostasis. Because alterations in intracellular signaling, including Ca^2+^-dependent pathways, contribute to cardiac remodeling and the development of left ventricular hypertrophy (LVH) [11,12,13,14], the cardiac response to sFB warrants investigation.

LVH is an important form of cardiac remodeling associated with conditions that increase cardiac workload, including hypertension and metabolic disorders such as obesity and diabetes [13,14,15]. In response to sustained hemodynamic stress, cardiomyocytes increase in size, leading to thickening of the left ventricular wall and changes in ventricular geometry. Although initially adaptive, persistent hypertrophic remodeling can become maladaptive and is associated with fibrosis, inflammation, cardiomyocyte dysfunction, and activation of cell-death pathways [13,14,15]. These changes increase the risk of adverse cardiovascular outcomes, including heart failure and arrhythmias [13,14,15].

Progressive cardiac remodeling can have systemic consequences that extend beyond the myocardium. Hemodynamic alterations associated with cardiac dysfunction may affect extracardiac organs, while pulmonary and venous congestion can contribute to changes in organs such as the lungs and liver [14,15,16,17]. These interactions illustrate the systemic consequences that can accompany pathological cardiac remodeling.

Current management of LVH primarily targets the underlying cause and may include angiotensin receptor blockers, β-blockers, diuretics, and other therapies aimed at reducing cardiac workload and controlling associated cardiovascular risk factors. Nevertheless, pathological cardiac remodeling may persist despite treatment, supporting continued investigation of additional strategies capable of modulating the hypertrophic response. In this context, boron-containing compounds have been proposed as potential modulators of processes associated with LVH [18].

Based on these observations, we hypothesized that sFB administration could modify the cardiac remodeling response induced by isoproterenol and that the biological response might depend on the timing of administration. Therefore, this study evaluated the short-term cardiac effects of sFB and compared the responses associated with its administration before and after induction of cardiac hypertrophy, with additional assessment of histological, molecular, and biochemical responses in the lung, intestine, kidney, and liver.

## 2. Results

### 2.1. Cardiac Effects of Short-Term sFB Administration

**Figure 2:**
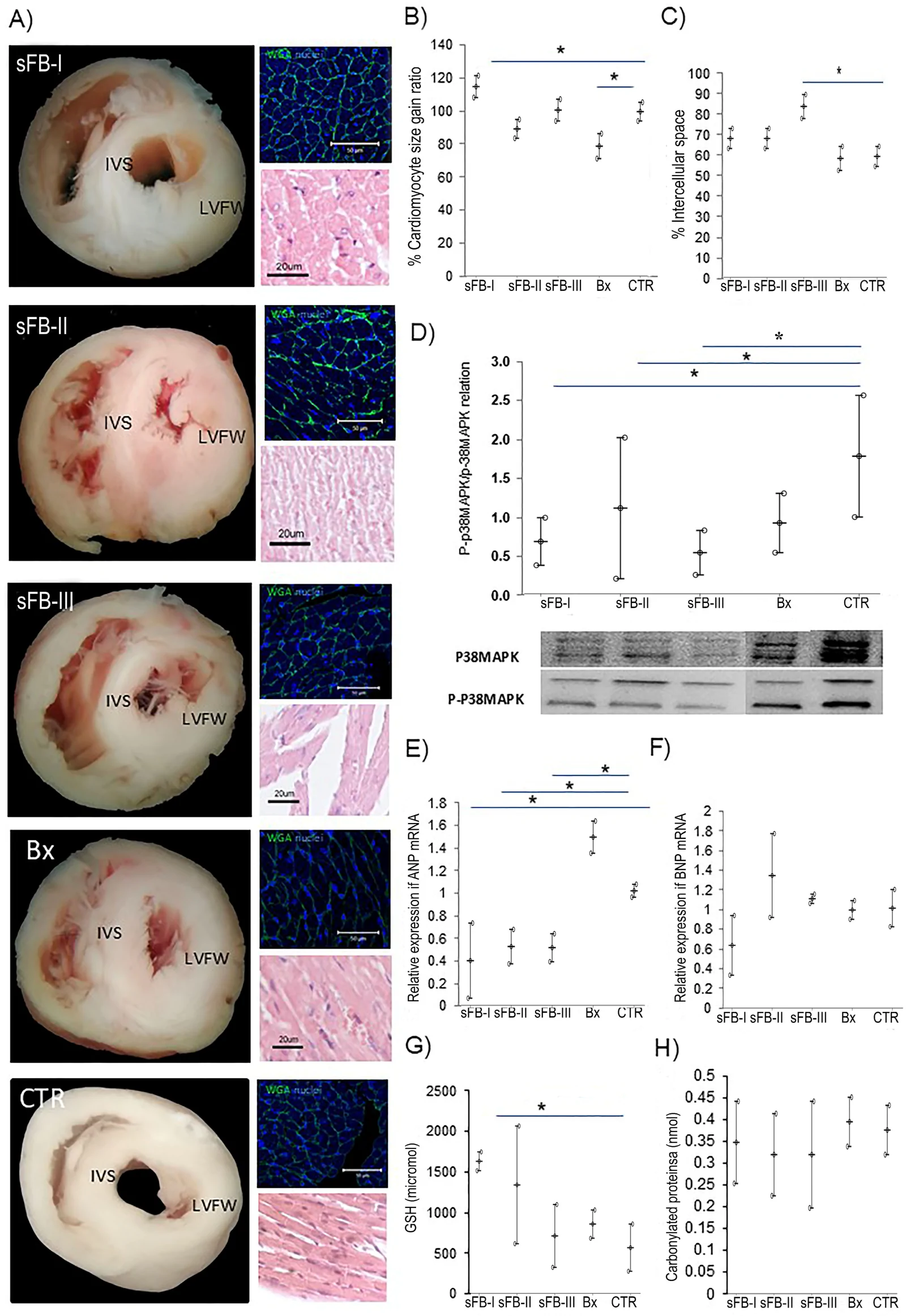
Cardiac effects of short-term synthetic fructoborate administration. (**A**) Representative heart cross-sections, hematoxylin and eosin (H&E)-stained sections, and wheat germ agglutinin (WGA)-stained sections from mice treated with 17 mg/kg sFB (sFB-I), 34 mg/kg sFB (sFB-II), 68 mg/kg sFB (sFB-III), 100 mg/kg sodium tetraborate (Bx), or vehicle (CTR). (**B**) Relative cardiomyocyte size. (**C**) Interstitial space. (**D**) p-p38 MAPK/p38 MAPK ratio determined by Western blot analysis. Relative mRNA levels of (**E**) *ANP* and (**F**) *BNP*. (**G**) Plasma GSH levels. (**H**) Plasma protein carbonyl levels. IVS, interventricular septum; LVFW, left ventricular free wall. Individual values are shown together with mean ± SD. Horizontal lines indicate the pairwise comparisons shown in the figure. Statistical analysis was performed using one-way ANOVA followed by Tukey’s post hoc test for multiple pairwise comparisons. * *p* < 0.05 was considered statistically significant.

We first examined the effects of sFB administration on cardiac structure by evaluating left ventricular muscle fiber organization, cardiomyocyte area, and interstitial space (Figure 2A). No fibrosis was detected following sFB administration. Cardiomyocyte area was significantly increased in mice treated with 17 mg/kg sFB (sFB-I), whereas no significant increase was observed at 34 or 68 mg/kg sFB (Figure 2B). Treatment with sodium tetraborate (Bx) reduced cardiomyocyte area relative to the control group. Mice treated with 68 mg/kg sFB (sFB-III) also showed a significant increase in interstitial space (*p* = 0.015) (Figure 2C). Overall, short-term sFB administration did not produce a consistent dose-dependent increase in cardiomyocyte area.

We next examined molecular markers associated with cardiac stress and remodeling. The p-p38 MAPK/p38 MAPK ratio was significantly lower in the hearts of mice treated with 17, 34, or 68 mg/kg sFB than in those of control mice (Figure 2D). *ANP* mRNA levels were significantly lower in all three sFB-treated groups than in the Bx group (Figure 2E), whereas *BNP* mRNA levels did not differ significantly among the experimental groups (Figure 2F). Overall, the molecular markers evaluated did not show a pattern consistent with increased cardiac stress across the sFB doses under these experimental conditions.

Finally, we examined plasma GSH and protein carbonyl levels as indicators of antioxidant status and oxidative protein damage, respectively (Figure 2G,H). GSH levels were significantly higher in mice treated with 17 mg/kg sFB (sFB-I) than in control mice (*p* = 0.029), whereas no significant difference was observed between the Bx and control groups (Figure 2G). Protein carbonyl levels did not differ significantly among the experimental groups (Figure 2H). Thus, the lowest sFB dose increased plasma GSH levels without detectable changes in protein carbonyl levels. Based on the overall structural, molecular, and biochemical profile observed after short-term administration, 34 mg/kg sFB was selected for subsequent experiments because it showed no major structural alterations and no significant changes in *BNP* mRNA or protein carbonyl levels relative to the control group.

### 2.2. Cardiac and Extracardiac Effects of sFB Administration in the Isoproterenol-Induced Cardiac Hypertrophy Model

**Figure 3:**
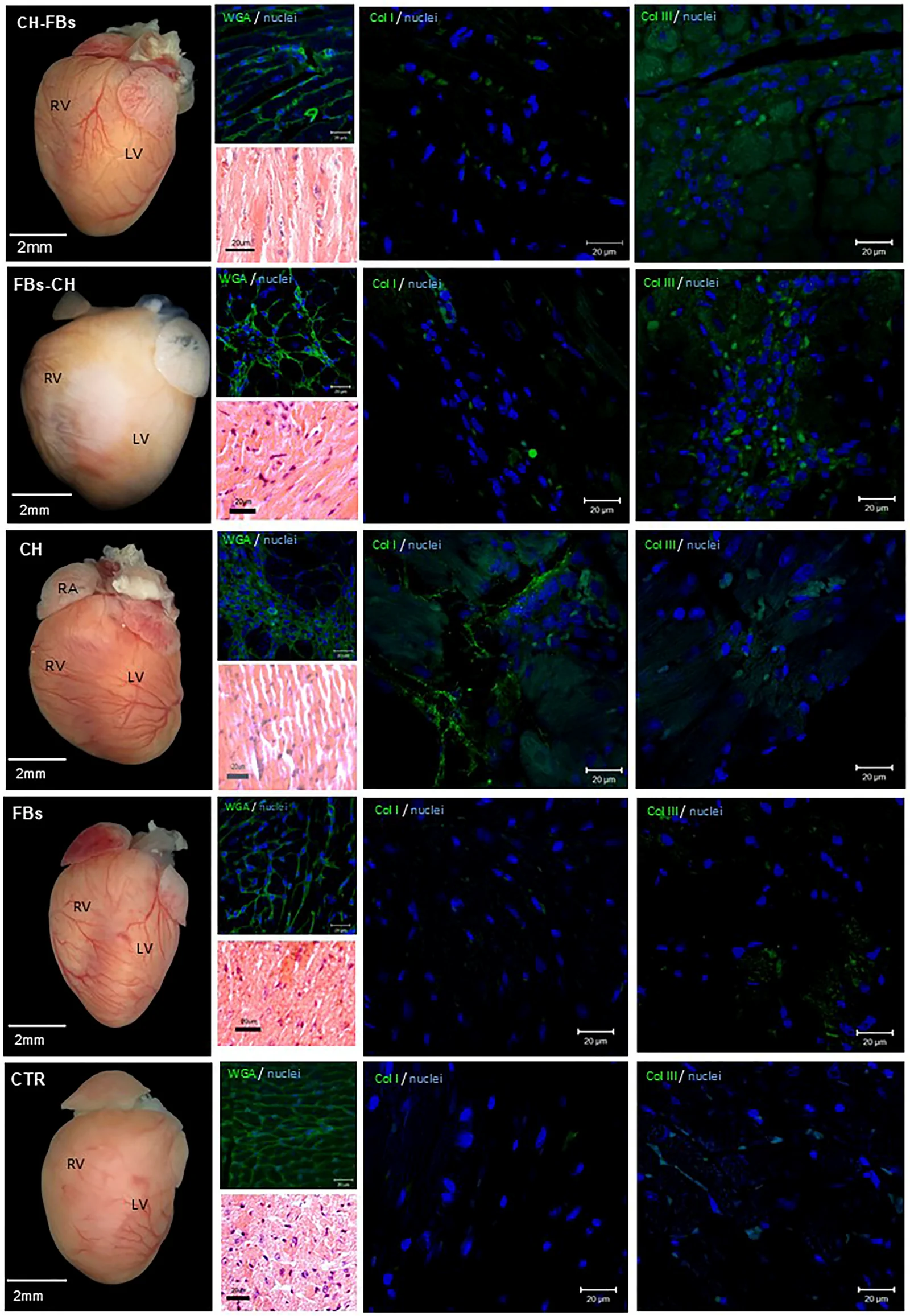
Representative cardiac histology and immunofluorescence in the experimental groups. Representative heart sections from mice receiving 34 mg/kg sFB after induction of cardiac hypertrophy (CH-sFB), 34 mg/kg sFB before induction of cardiac hypertrophy (sFB-CH), isoproterenol alone (CH), sFB alone (sFB), or vehicle (CTR). Cardiac morphology was evaluated by hematoxylin and eosin (H&E) staining. Cardiomyocyte membranes were visualized by wheat germ agglutinin (WGA) staining, and collagen I and III deposition was evaluated by immunofluorescence and confocal microscopy. Nuclei were counterstained in blue.

**Figure 4:**
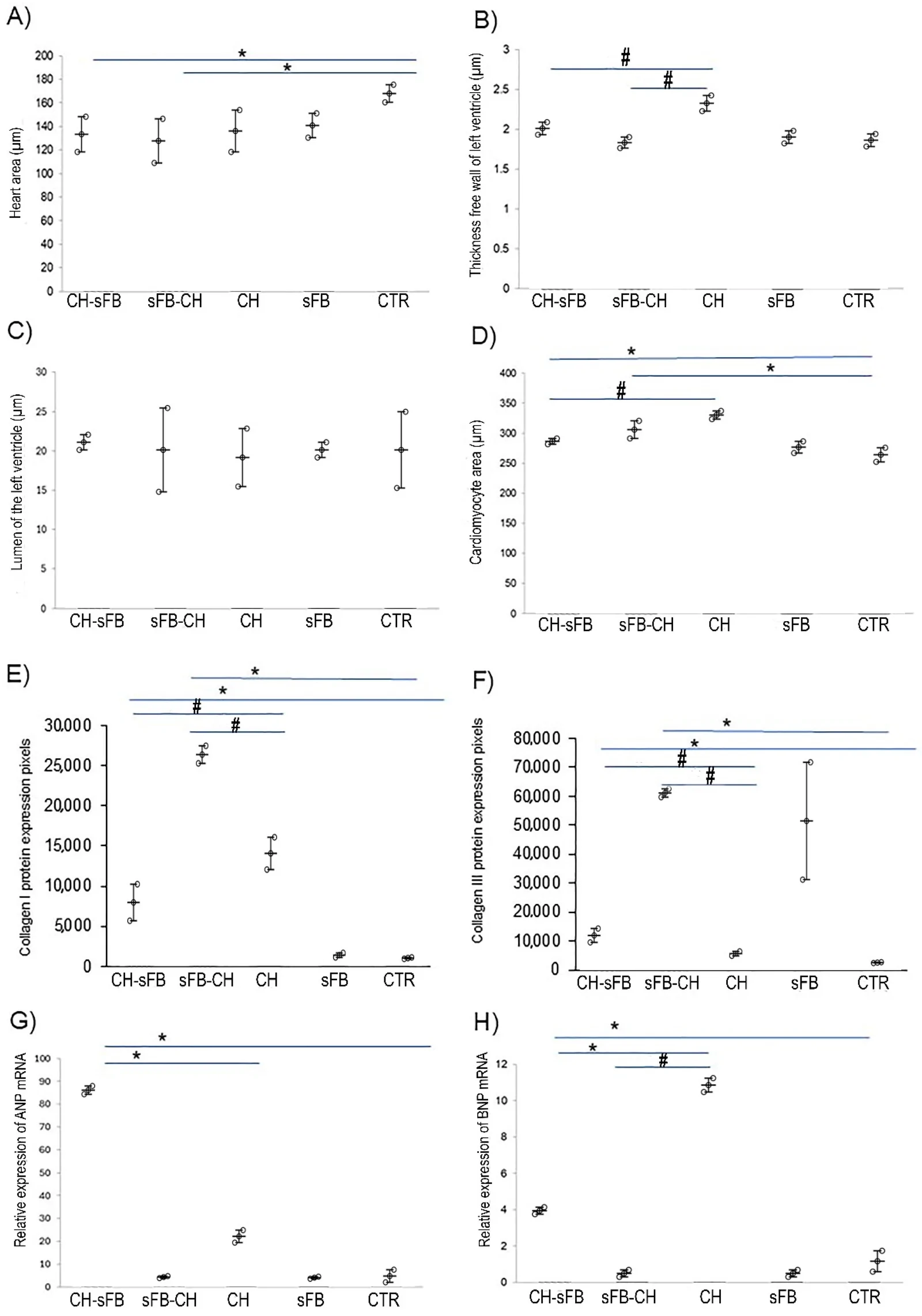
Cardiac structural and molecular changes following sFB administration in the isoproterenol-induced cardiac hypertrophy model. (**A**) Ventricular cross-sectional area. (**B**) Left ventricular free-wall thickness. (**C**) Left ventricular lumen area. (**D**) Cardiomyocyte area. Quantification of (**E**) collagen I and (**F**) collagen III staining. Relative mRNA levels of (**G**) ANP and (**H**) BNP. Individual values are shown together with mean ± SD. Horizontal lines indicate the pairwise comparisons shown in the figure. Statistical analysis was performed using one-way ANOVA followed by Tukey’s post hoc test for multiple pairwise comparisons. A *p* value < 0.05 was considered statistically significant. * *p* < 0.05 vs. CTR; # *p* < 0.05 vs. CH. CTR, control; sFB, sFB-treated control group; CH, isoproterenol-induced cardiac hypertrophy; CH-sFB, sFB administered after induction of cardiac hypertrophy; sFB-CH, sFB administered before induction of cardiac hypertrophy.

We first examined the effects of sFB administration after cardiac hypertrophy induction on ventricular structure (Figure 3). Heart cross-sectional area did not differ significantly between the CH-sFB and untreated CH groups (Figure 4A). However, LV free-wall thickness was significantly lower in the CH-sFB group than in the CH group (*p* = 0.015), whereas ventricular lumen area was significantly greater (*p* = 0.013) (Figure 4B,C). Cardiomyocyte area was also significantly lower following sFB treatment (*p* = 0.003), although it remained greater than that observed in control mice (Figure 4D). Collagen I and III deposition was significantly lower in the CH-sFB group than in the untreated CH group (*p* = 0.015) (Figure 4E,F). Together, these findings indicate that sFB administration after the induction of cardiac hypertrophy partially attenuated the structural remodeling associated with isoproterenol treatment.

**Figure 5:**
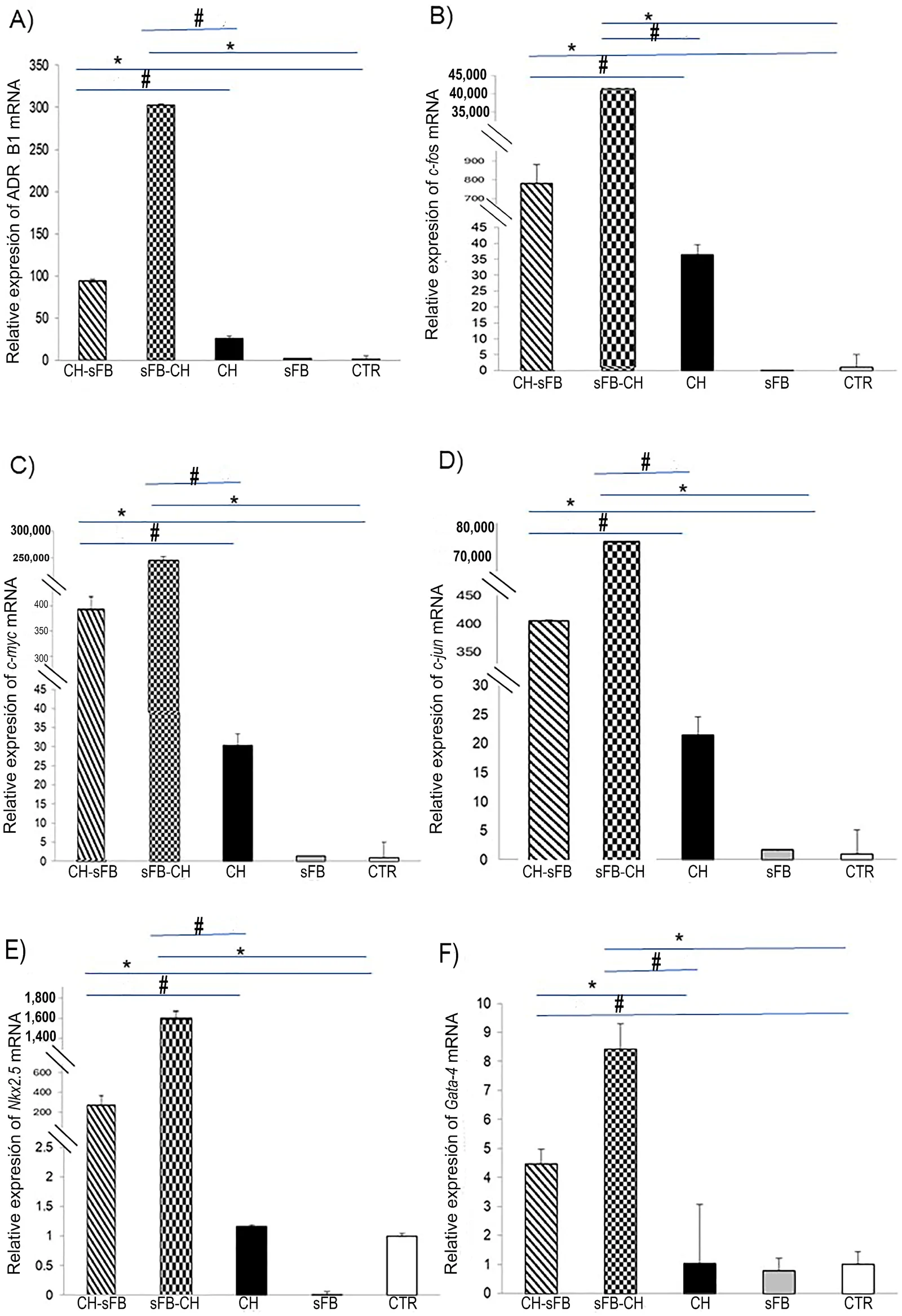
Cardiac expression of early-response and hypertrophy-associated genes following sFB administration in the isoproterenol-induced cardiac hypertrophy model. Relative cardiac mRNA levels of (**A**) *ADRB1*, (**B**) *c-Fos*, (**C**) *c-Myc*, (**D**) *c-Jun*, (**E**) *Nkx2.5*, and (**F**) *Gata4* in mice receiving sFB after induction of cardiac hypertrophy (CH-sFB), sFB before induction of cardiac hypertrophy (sFB-CH), isoproterenol alone (CH), sFB alone (sFB), or vehicle (CTR). Data are presented as mean ± SD. Statistical analysis was performed using one-way ANOVA followed by Tukey’s post hoc test for multiple pairwise comparisons. A *p* value < 0.05 was considered statistically significant. * *p* < 0.05 vs. CTR; # *p* < 0.05 vs. CH. CTR, control; sFB, sFB-treated control group; CH, isoproterenol-induced cardiac hypertrophy; CH-sFB, sFB administered after induction of cardiac hypertrophy; sFB-CH, sFB administered before induction of cardiac hypertrophy.

The molecular response showed a different pattern. *BNP* mRNA levels were lower in CH-sFB mice than in untreated CH mice, whereas *ANP* mRNA levels were higher in the CH-sFB group (Figure 4G,H). Thus, the structural changes observed following sFB treatment were accompanied by differential regulation of these cardiac remodeling markers. We next examined the cardiac expression of genes associated with early-response and hypertrophic signaling (Figure 5). Compared with the untreated CH group, CH-sFB mice showed significantly higher mRNA levels of the *β1-adrenergic receptor* (*ADRB1*; *p* = 0.047), *c-jun* (*p* = 0.047), *c-fos* (*p* = 0.015), and *c-myc* (*p* = 0.015). The mRNA levels of the cardiac transcription factors *Nkx2.5* (*p* = 0.047) and *Gata-4* (*p* = 0.047) were also significantly higher in the CH-sFB group. Notably, the sFB-CH group showed a pronounced expression profile for these genes, indicating a distinct transcriptional response when sFB was administered before rather than after induction of cardiac hypertrophy. In contrast, mice receiving sFB alone showed low expression levels of these markers, generally comparable to those observed in control mice. Together, these findings indicate that the structural changes associated with sFB administration were accompanied by a marked, timing-dependent cardiac transcriptional response.

**Figure 6:**
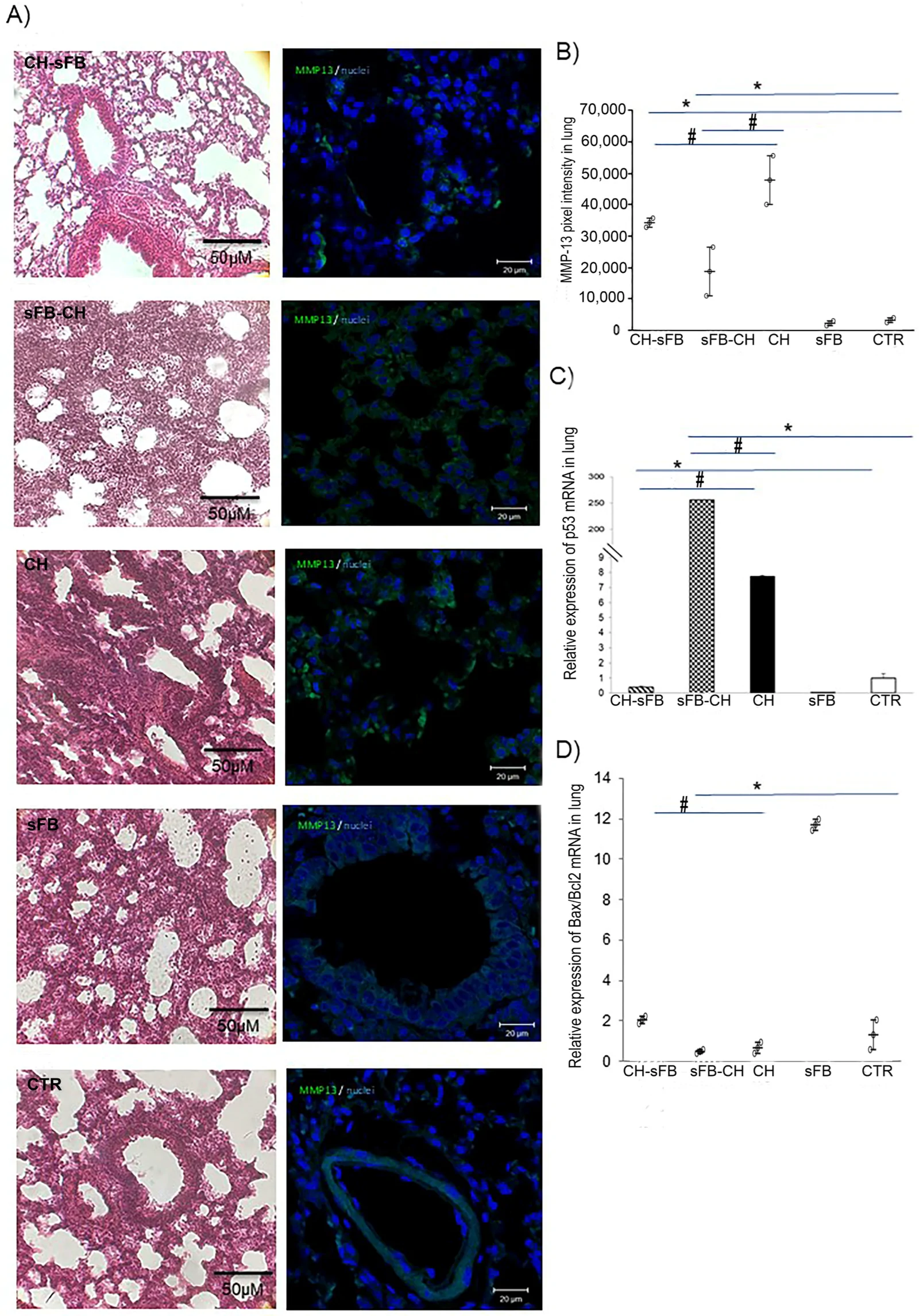
Pulmonary changes associated with sFB administration in the isoproterenol-induced cardiac hypertrophy model. (**A**) Representative lung sections from mice receiving 34 mg/kg sFB after induction of cardiac hypertrophy (CH-sFB), 34 mg/kg sFB before induction of cardiac hypertrophy (sFB-CH), isoproterenol alone (CH), sFB alone (sFB), or vehicle (CTR). Lung morphology was evaluated by hematoxylin and eosin (H&E) staining, and MMP-13 was evaluated by immunofluorescence. (**B**) Quantification of MMP-13 immunofluorescence intensity. (**C**) Relative pulmonary *p53* mRNA levels. (**D**) Pulmonary *Bax/Bcl*-2 mRNA ratio. For panels (**B**,**D**), individual values are shown together with mean ± SD; panel C is presented as mean ± SD. Statistical analysis was performed using one-way ANOVA followed by Tukey’s post hoc test for multiple pairwise comparisons. A *p* value < 0.05 was considered statistically significant. * *p* < 0.05 vs. CTR; # *p* < 0.05 vs. CH. CTR, control; sFB, sFB-treated control group; CH, isoproterenol-induced cardiac hypertrophy; CH-sFB, sFB administered after induction of cardiac hypertrophy; sFB-CH, sFB administered before induction of cardiac hypertrophy.

We next examined pulmonary changes associated with sFB administration after the induction of cardiac hypertrophy (Figure 6). Histological examination of the CH-sFB group showed less extensive parenchymal alterations and limited peribronchial inflammatory infiltrates compared with the untreated CH group. However, alveolar septal thickening remained evident, indicating persistent alterations in lung architecture (Figure 6A). MMP-13 immunofluorescence intensity was lower in the CH-sFB group than in the CH group (*p* = 0.015), although it remained above the levels observed in the sFB and control groups (Figure 6B). Pulmonary *p53* mRNA levels were also significantly lower in CH-sFB mice than in untreated CH mice (*p* = 0.015) (Figure 6C). In contrast, the *Bax/Bcl-2* mRNA ratio was higher in the CH-sFB group than in the CH group (*p* = 0.015) (Figure 6D). Together, these findings indicate that sFB administration after induction of cardiac hypertrophy was associated with partial changes in the pulmonary response, without complete restoration of lung architecture or normalization of the molecular markers evaluated.

Notably, mice receiving sFB alone showed a marked increase in the pulmonary *Bax/Bcl-2* mRNA ratio compared with control mice (Figure 6D). Histological examination also showed alveolar septal thickening, increased cellularity, inflammatory infiltrates, and a moderate reduction in airspace. These findings indicate that short-term sFB administration in the absence of cardiac hypertrophy was associated with pulmonary changes that warrant further investigation.

**Figure 7:**
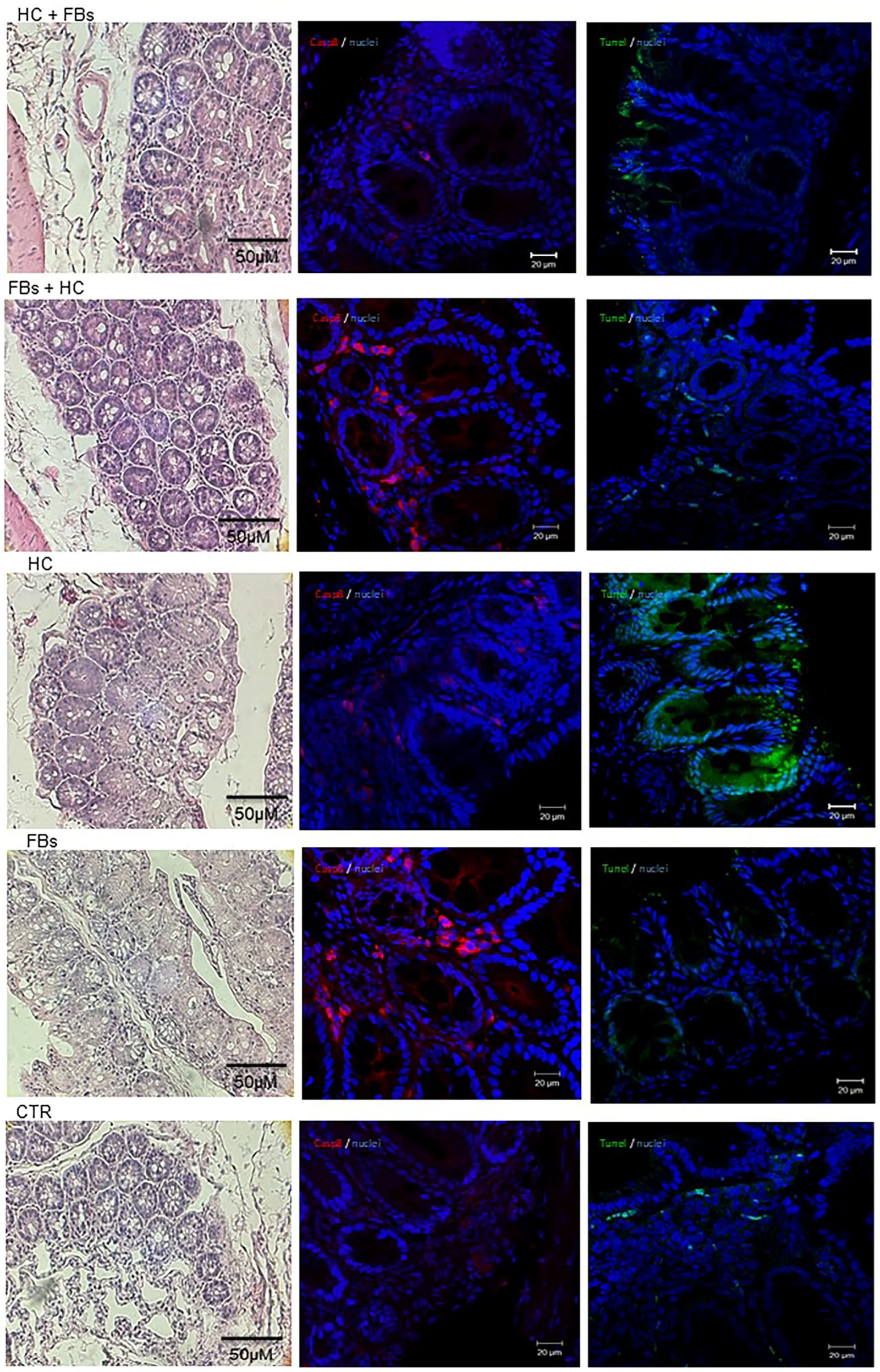
Intestinal histology and cell-death-associated markers following sFB administration in the isoproterenol-induced cardiac hypertrophy model. Representative intestinal sections from mice receiving 34 mg/kg sFB after induction of cardiac hypertrophy (CH-sFB), 34 mg/kg sFB before induction of cardiac hypertrophy (sFB-CH), isoproterenol alone (CH), sFB alone (sFB), or vehicle (CTR). Tissue morphology was evaluated by hematoxylin and eosin (H&E) staining. Caspase-8 was detected by immunofluorescence (red), and DNA fragmentation was evaluated by TUNEL staining (green). Nuclei were counterstained with DRAQ7 (blue).

**Figure 8:**
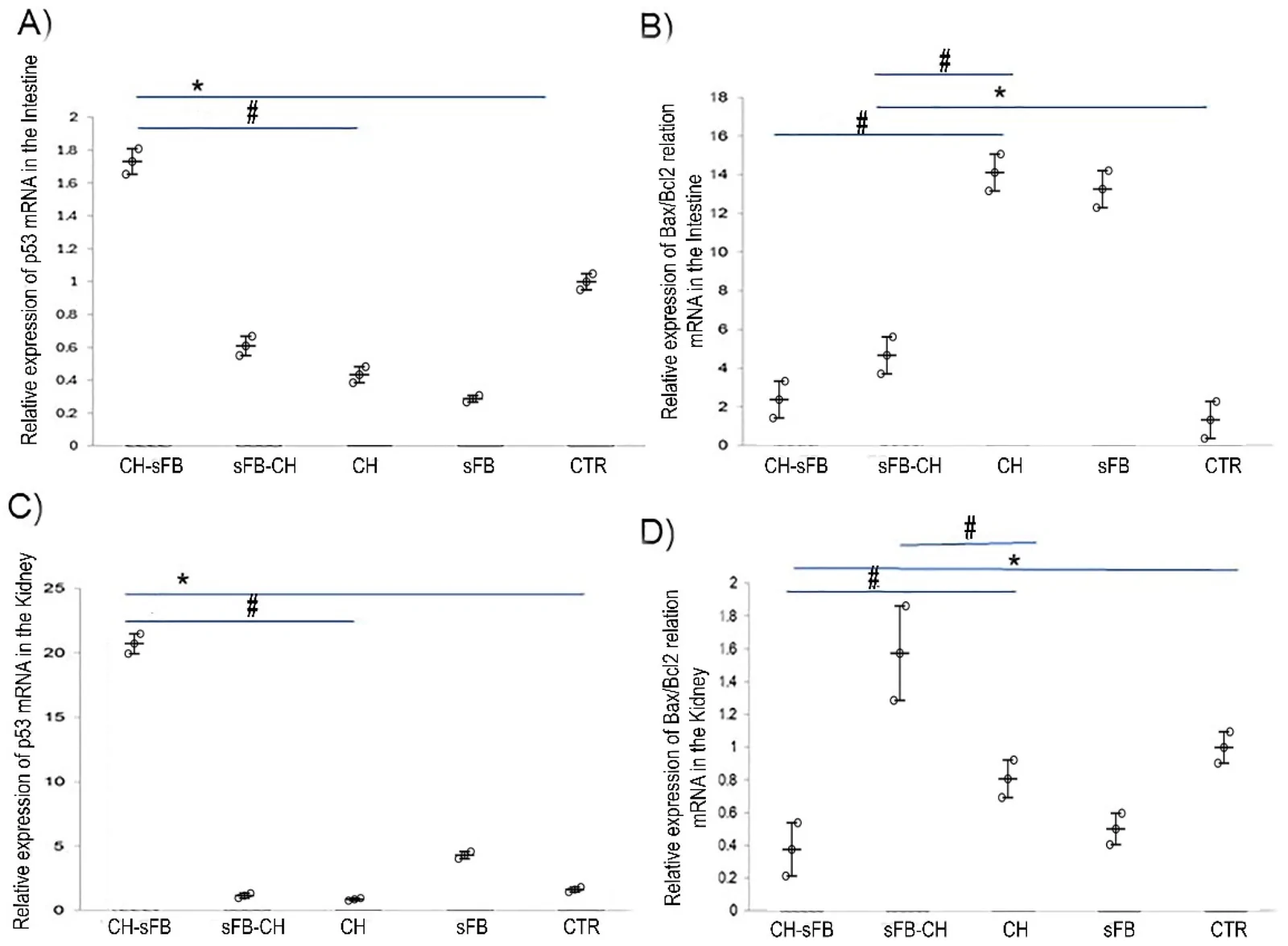
Expression of cell-death-associated markers in the intestine and kidney following sFB administration in the isoproterenol-induced cardiac hypertrophy model. Relative *p53* mRNA levels and *Bax/Bcl-2* mRNA ratios in the (**A**,**B**) ileum and (**C**,**D**) kidney of mice receiving sFB after induction of cardiac hypertrophy (CH-sFB), sFB before induction of cardiac hypertrophy (sFB-CH), isoproterenol alone (CH), sFB alone (sFB), or vehicle (CTR). Gene expression was normalized to GAPDH. Individual values are shown together with mean ± SD. Statistical analysis was performed using one-way ANOVA followed by Tukey’s post hoc test for multiple pairwise comparisons. A *p* value < 0.05 was considered statistically significant. * *p* < 0.05 vs. CTR; # *p* < 0.05 vs. CH. CTR, control; sFB, sFB-treated control group; CH, isoproterenol-induced cardiac hypertrophy; CH-sFB, sFB administered after induction of cardiac hypertrophy; sFB-CH, sFB administered before induction of cardiac hypertrophy.

In the intestine, histological examination of the CH-sFB group showed persistent alterations in tissue architecture, although the crypts exhibited a more regular morphology than those observed in the untreated CH group (Figure 7). Areas of increased cellularity and thickening of the surrounding tissue remained evident. Caspase-8 immunofluorescence was lower in the CH-sFB group than in the CH group, and TUNEL staining was also reduced (Figure 7). At the molecular level, intestinal *p53* mRNA levels were significantly higher in CH-sFB mice than in untreated CH mice (*p* = 0.047), whereas the *Bax/Bcl*-2 mRNA ratio was significantly lower (*p* = 0.015) (Figure 8A,B). Together, these findings indicate that sFB administration after induction of cardiac hypertrophy was associated with partial histological changes and a distinct cell-death-related response in the intestine. Mice receiving sFB alone showed a high intestinal *Bax/Bcl-2* mRNA ratio, suggesting that the intestinal response to sFB may depend on the underlying physiological context.

In the kidney, *p53* mRNA levels were significantly higher in the CH-sFB group than in the untreated CH group (*p* = 0.015) (Figure 8C). In contrast, the *Bax/Bcl-2* mRNA ratio was significantly lower in CH-sFB mice than in CH mice (*p* = 0.015) (Figure 8D). The sFB-CH group showed low *p53* mRNA levels together with a higher *Bax/Bcl-2* mRNA ratio, whereas mice receiving sFB alone showed a distinct pattern of *p53* and *Bax/Bcl-2* mRNA expression (Figure 8C,D).

**Figure 9:**
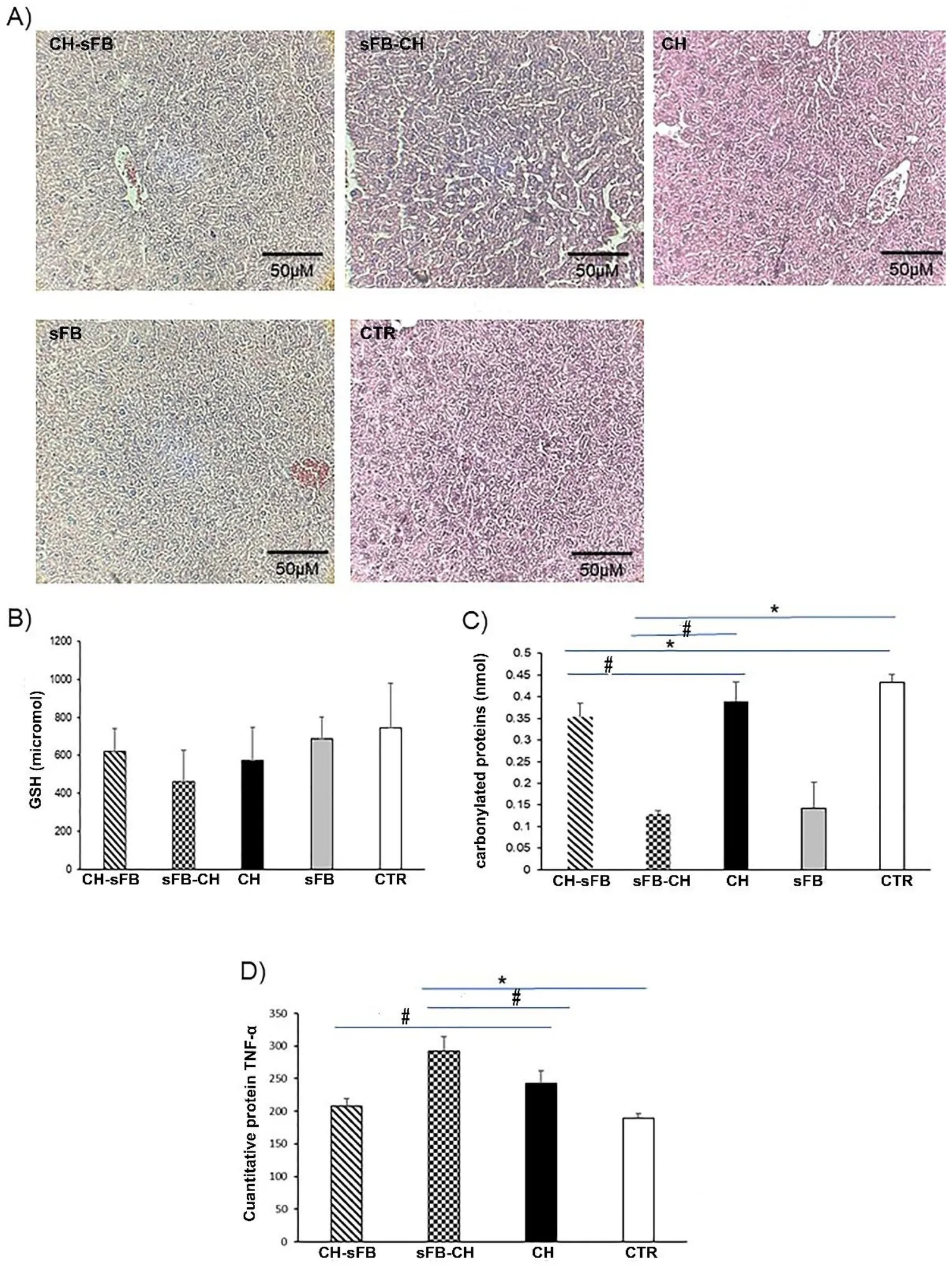
Hepatic histology and circulating biochemical markers associated with sFB administration in the isoproterenol-induced cardiac hypertrophy model. (**A**) Representative H&E-stained liver sections from mice receiving 34 mg/kg sFB after induction of cardiac hypertrophy (CH-sFB), 34 mg/kg sFB before induction of cardiac hypertrophy (sFB-CH), isoproterenol alone (CH), sFB alone (sFB), or vehicle (CTR). (**B**) Plasma GSH levels. (**C**) Plasma protein carbonyl levels. (**D**) Plasma TNF-α levels. Data are presented as mean ± SD. Statistical analysis was performed using one-way ANOVA followed by Tukey’s post hoc test for multiple pairwise comparisons. A *p* value < 0.05 was considered statistically significant. * *p* < 0.05 vs. CTR; # *p* < 0.05 vs. CH. CTR, control; sFB, sFB-treated control group; CH, isoproterenol-induced cardiac hypertrophy; CH-sFB, sFB administered after induction of cardiac hypertrophy; sFB-CH, sFB administered before induction of cardiac hypertrophy.

Finally, we examined hepatic changes following sFB administration in the isoproterenol-induced cardiac hypertrophy model (Figure 9). Histological examination of the CH-sFB group showed persistent alterations in hepatic architecture, including areas of focal tissue damage and inflammatory infiltrates (Figure 9A). GSH levels did not differ significantly between the CH-sFB and untreated CH groups, and protein carbonyl levels were also similar between these groups (Figure 9B,C). Thus, the biochemical markers evaluated did not provide evidence of a clear improvement in hepatic oxidative status following sFB administration after induction of cardiac hypertrophy.

Mice receiving sFB alone showed mild histological alterations, including some disorganization of hepatic cords and small inflammatory foci (Figure 9A). Protein carbonyl levels were lower in the sFB group than in the CH group, whereas GSH levels did not differ significantly from those in control mice (Figure 9B,C). Finally, plasma TNF-α levels were significantly lower in CH-sFB mice than in untreated CH mice (*p* = 0.029) (Figure 9D). Overall, these findings indicate that hepatic and systemic responses associated with sFB varied by experimental condition.

### 2.3. Effects of sFB Administration Before Induction of Cardiac Hypertrophy

We next examined the effects of sFB administration before induction of cardiac hypertrophy (sFB-CH). Heart cross-sectional area was similar in the sFB-CH and CH groups, whereas LV free-wall thickness and ventricular lumen area in sFB-CH mice were comparable to those observed in control mice (Figure 4A–C). Cardiomyocyte area was significantly greater in the sFB-CH group than in the control group (*p* = 0.007), although it remained lower than in untreated CH mice (Figure 4D). In contrast, collagen I and III deposition was markedly increased in the sFB-CH group (Figure 4E,F). *ANP* and *BNP* mRNA levels were low in sFB-CH mice compared with the untreated CH group (Figure 4G,H). Thus, sFB administration before induction of cardiac hypertrophy was associated with differential effects across structural and molecular markers of cardiac remodeling.

A marked transcriptional response was also observed in the sFB-CH group. Cardiac mRNA levels of ADRB1, *c-Fos*, *c-Myc*, *c-Jun*, *Nkx2.5*, and *Gata4* were significantly higher than those observed in the untreated CH and control groups (Figure 5A–F). These findings indicate that, despite the comparatively preserved LV wall thickness and lumen area, sFB administration before isoproterenol exposure did not prevent the molecular and extracellular-matrix changes associated with the hypertrophic response.

Pulmonary histology in the sFB-CH group showed alterations in alveolar architecture, including irregular and relatively small airspaces and disorganization of the alveolar structure (Figure 6A). MMP-13 immunofluorescence intensity was significantly lower in sFB-CH mice than in untreated CH mice (*p* = 0.015) (Figure 6B). In contrast, pulmonary *p53* mRNA levels were significantly higher in the sFB-CH group than in the CH group (*p* = 0.015), whereas the *Bax/Bcl-2* mRNA ratio did not differ significantly between these groups (Figure 6C,D). Thus, sFB administration before induction of cardiac hypertrophy was associated with persistent histological alterations and a distinct pulmonary molecular response.

In the intestine, the overall tissue architecture of sFB-CH mice appeared relatively preserved, although increased cellularity remained evident in the lamina propria (Figure 7). Caspase-8 immunofluorescence was detected in the intestinal tissue, whereas TUNEL staining appeared lower than in the untreated CH group (Figure 7). Intestinal *p53* mRNA levels did not differ significantly between the sFB-CH and CH groups (Figure 8A). The *Bax/Bcl-2* mRNA ratio showed a lower value in sFB-CH mice than in untreated CH mice (Figure 8B). Together, these findings indicate that sFB administration before induction of cardiac hypertrophy was associated with a distinct intestinal histological and cell-death-related response.

In the kidney, *p53* mRNA levels in the sFB-CH group did not differ significantly from those in the untreated CH group (Figure 8C). In contrast, the *Bax/Bcl-2* mRNA ratio was significantly higher in sFB-CH mice than in control mice (*p* < 0.001) (Figure 8D). These findings indicate that sFB administration before induction of cardiac hypertrophy was associated with a distinct pattern of renal cell-death-associated markers.

In the liver, the sFB-CH group showed marked disruption of the hepatic architecture, with areas of focal tissue damage and dense inflammatory infiltrates (Figure 9A). Disorganization of the hepatic cords and thickening of the surrounding tissue were also evident. GSH and protein carbonyl levels showed lower numerical values in the sFB-CH group than in the control and CH groups, respectively (Figure 9B,C). Plasma TNF-α levels were elevated in sFB-CH mice and were higher than those observed in the other experimental groups (Figure 9D). Together, these findings indicate that sFB administration before induction of cardiac hypertrophy did not prevent the hepatic alterations associated with this experimental condition.

## 3. Discussion

The present study shows that the biological responses associated with sFB differ according to the experimental context and timing of administration. Short-term administration of sFB at the doses evaluated did not produce a consistent pattern of cardiac structural or molecular alterations indicative of overt cardiac injury. In the isoproterenol-induced cardiac hypertrophy model, sFB administration after hypertrophy induction was associated with attenuation of several structural features of cardiac remodeling, whereas administration before isoproterenol exposure produced a different response, with persistent extracellular-matrix and transcriptional changes. Extracardiac responses were also heterogeneous across the lung, intestine, kidney, and liver.

### 3.1. Short-Term Effects of Synthetic Fructoborate

Boron is a micronutrient widely present in food and drinking water and has been associated with several physiological processes in mammals [19]. Experimental and nutritional studies have suggested that inadequate boron intake may affect bone metabolism, cognitive function, and steroid hormone levels [20]. However, the biological and toxicological effects of boron can vary according to its chemical form, dose, route of administration, and duration of exposure. Adverse effects have been reported following exposure to high doses of some boron-containing compounds [7].

Previous studies of sFB provide a useful context for the doses evaluated in the present study. Córdoba-Chávez et al. [10] reported acute effects following intraperitoneal administration of substantially higher doses of sFB, including toxicity at 1265 mg/kg and sedation at 531 mg/kg without mortality. Valenzuela-Schejtman et al. [21] evaluated intraperitoneal administration of 51.9 mg/kg sFB in an experimental seizure model and reported no changes in seizure latency or maximal seizure phase, together with reduced neuronal loss. Nevertheless, differences in administration route and experimental model limit direct comparison with the present study.

In our short-term administration experiment, sFB at 17, 34, and 68 mg/kg did not produce a consistent dose-dependent increase in cardiomyocyte area, although interstitial space was increased at the highest dose. Moreover, the *p*-p38 MAPK/p38 MAPK ratio was lower in sFB-treated mice than in control mice, whereas *BNP* mRNA levels did not differ significantly among groups. *ANP* mRNA levels also showed no pattern consistent with increasing cardiac stress across the sFB doses evaluated. Together, these findings indicate that short-term sFB administration did not produce a consistent pattern of structural and molecular changes indicative of overt cardiac injury under the experimental conditions evaluated.

The biological effects of boron-containing compounds can vary according to their chemical structure, which may influence their interactions with biological targets and their effects on processes such as oxidative stress and inflammation. In the present study, short-term administration of 17 mg/kg sFB significantly increased plasma GSH levels, whereas higher sFB doses did not produce a significant increase. Protein carbonyl levels remained unchanged across the sFB doses evaluated. Previous studies with other boron-containing compounds have also reported changes in antioxidant markers. For example, Ince et al. [22] reported increased blood GSH and plasma vitamin C levels, along with reduced lipid peroxidation, following borax administration. Although the compound and experimental conditions differ from those used here, these observations support further investigation of the relationship between boron-containing compounds and redox homeostasis.

Within this context, calcium fructoborate (CaFB), a naturally occurring sugar–borate ester, represents a particularly relevant comparator because it has been investigated for its effects on inflammatory and cardiovascular-related biomarkers [5,18]. Other boron-containing compounds, including boric acid and borates, have also been associated with modulation of oxidative and metabolic processes [7,22]. However, these compounds are not direct structural equivalents of the sFB evaluated here, and differences in boron speciation, carbohydrate environment, and molecular structure may substantially influence their biological activity. Therefore, the changes in GSH and other biochemical responses observed in the present study cannot be attributed to boron alone. Comparative studies including sFB, structurally related boron–carbohydrate adducts [10], and inorganic boron compounds will be necessary to determine the extent to which the observed responses are associated with the intact sFB structure or with boron-containing species generated after administration.

The renal molecular response to sFB was more complex. Changes in p53 mRNA expression and the *Bax/Bcl-2* mRNA ratio differed according to the experimental condition, indicating that these markers should not be interpreted as direct evidence of either renal protection or toxicity. *p53* has context-dependent functions in cellular stress responses, including cell-cycle arrest, DNA repair, metabolic adaptation, and, under conditions of sustained or severe damage, induction of cell death pathways [23]. Accordingly, the discordance observed between *p53* mRNA expression and the *Bax/Bcl-2* mRNA ratio in our study may reflect differences in stress-response pathways; however, the present data are insufficient to establish whether these changes represent adaptation, apoptosis, or toxicity. Assessment at the protein level, together with renal histopathology and functional markers, would be required to distinguish among these possibilities.

Previous studies further suggest that renal responses to boron-containing compounds depend on dose and experimental context. Experimental studies with boric acid have reported both potentially beneficial and adverse renal responses. In ostrich chicks, low boron doses were associated with improved antioxidant capacity and reduced apoptosis, whereas higher doses produced adverse renal effects [24]. In a rat model of acute kidney injury, boric acid produced context-dependent responses, and its combination with conivaptan was associated with increased BUN and creatinine levels, highlighting the importance of treatment conditions and possible interactions [25]. In addition, observational data in kidney transplant recipients showed that higher boron exposure was associated with lower mortality, although these findings do not demonstrate a direct protective effect of boron on renal tissue [26]. These findings cannot be directly extrapolated to sFB but underscore the importance of evaluating renal effects specifically for this compound.

### 3.2. Effects of sFB on Isoproterenol-Induced Cardiac Hypertrophy

A notable finding of this study was the dissociation between structural and molecular responses following sFB administration after induction of cardiac hypertrophy. Compared with untreated hypertrophic mice, CH-sFB mice showed reduced left ventricular wall thickness and cardiomyocyte area, increased ventricular lumen area, and lower collagen I and III deposition, supporting a partial attenuation of cardiac structural remodeling. However, these structural changes were not accompanied by uniform normalization of the molecular markers evaluated. This apparent dissociation may reflect the complex and potentially asynchronous nature of structural and molecular remodeling after cardiac injury [13,14,27], although the present study was not designed to establish the mechanisms underlying these differences.

A similar lack of uniformity was observed in the extracardiac tissues evaluated. Although sFB administration after hypertrophy induction was associated with attenuation of several cardiac structural changes, the lung, intestine, kidney, and liver exhibited distinct histological and molecular response patterns. In particular, *p53* expression and the Bax/Bcl-2 mRNA ratio varied among tissues and experimental conditions. These findings indicate that the extracardiac response to sFB cannot be interpreted as a uniform protective effect and suggest that tissue-specific responses should be considered when evaluating the systemic effects of sFB in this model.

The effects of sFB differed when the compound was administered before induction of cardiac hypertrophy. In the sFB-CH group, left ventricular wall thickness and lumen area were comparable to those observed in control mice, whereas cardiomyocyte area remained significantly greater than in controls but lower than in untreated hypertrophic mice. These apparently favorable structural findings contrasted with increased collagen I and III deposition and a marked transcriptional response involving *ADRB1*, *c-Fos*, *c-Myc*, *c-Jun*, *Nkx2.5*, and *Gata4*. Thus, administration of sFB before isoproterenol exposure did not uniformly prevent the structural and molecular changes associated with cardiac hypertrophy.

The extracardiac findings showed a similarly heterogeneous pattern. Lung, intestinal, renal, and hepatic responses differed across the histological and molecular markers evaluated, and the marked hepatic alterations observed in sFB-CH mice argue against interpreting these findings as evidence of generalized multi-organ protection. Taken together, the differences between the CH-sFB and sFB-CH groups suggest that the biological response to sFB in this model may vary, at least in part, according to the timing of administration relative to isoproterenol-induced cardiac injury.

The different responses observed between post-induction treatment and pretreatment suggest that the response associated with sFB may vary according to the stage of the remodeling process at which it is administered. The more consistent structural response observed when sFB was administered after hypertrophy induction, together with the lower collagen I and III deposition, raises the possibility of an association between sFB administration and processes involved in established extracellular-matrix remodeling. In contrast, pretreatment did not prevent collagen deposition and was accompanied by increased expression of several remodeling-associated genes, showing that these molecular responses remained detectable after isoproterenol exposure. Oxidative and inflammatory processes may contribute to these differences; however, the present GSH, protein carbonyl, and TNF-α data are insufficient to establish their involvement or causality [13,14]. Likewise, although calcium-dependent signaling contributed to the rationale for investigating sFB, calcium handling was not directly measured. Therefore, the mechanisms underlying the timing-dependent response to sFB remain to be established.

Several pathways not evaluated in the present study could contribute to the differences in cardiac structural remodeling observed across the experimental conditions. TGF-β/Smad signaling and the balance between extracellular-matrix synthesis and degradation are central regulators of cardiac fibrosis and could be relevant to the differences in collagen deposition observed between treatment conditions [28,29,30,31]. Oxidative-stress-responsive and inflammatory pathways may also contribute to pathological cardiac remodeling [13,14,30,31], although the circulating GSH, protein carbonyl, and TNF-α measurements obtained here do not establish their involvement in cardiac tissue. In addition, calcium-dependent signaling, including calcineurin/NFAT signaling and proteins involved in intracellular Ca^2+^ handling, warrants further investigation given its established role in hypertrophic signaling [13,14,32,33]. These pathways were not directly evaluated in the present study and should therefore be considered mechanistic hypotheses for future investigation rather than mechanisms of sFB action demonstrated by our data.

### 3.3. Extracardiac Responses to sFB Administration

The gut–heart axis and the integrity of the intestinal barrier have received increasing attention because of their potential involvement in cardiometabolic disease [34,35]. In the present study, intestinal histology and cell-death-associated markers differed according to the timing of sFB administration. Administration after induction of cardiac hypertrophy was associated with changes in intestinal architecture and reduced TUNEL staining relative to the untreated hypertrophic group, whereas administration before isoproterenol exposure produced a different molecular profile. Because intestinal permeability, epithelial junction proteins, and microbiota composition were not evaluated, the present findings cannot establish whether sFB directly affects intestinal barrier function or the gut–heart axis.

The extracardiac responses to sFB were heterogeneous and should not be interpreted as evidence of uniform multiorgan protection. In the kidney, post-induction sFB administration was associated with increased *p53* mRNA expression but a decreased Bax/Bcl-2 mRNA ratio. These apparently divergent changes may reflect the context-dependent functions of p53 in cellular stress responses, which include cell-cycle regulation, DNA repair, metabolic adaptation, and apoptosis [23]. However, neither *p53* expression nor the *Bax/Bcl-2* mRNA ratio alone is sufficient to establish whether the renal response was adaptive, cytoprotective, or potentially adverse. Additional protein-level analyses, direct assessment of cell death, and renal functional measurements would be required to distinguish among these possibilities.

A similarly cautious interpretation is warranted for the other extracardiac tissues. Some pulmonary and intestinal endpoints showed changes after post-induction sFB administration, but tissue architecture and molecular markers were not uniformly restored. Moreover, alterations were also observed in some tissues from mice receiving sFB alone. The hepatic response was particularly heterogeneous, with persistent histological abnormalities and no consistent improvement in the oxidative-status markers evaluated. Collectively, these findings indicate that the extracardiac responses associated with sFB varied across tissues and experimental conditions and included both potentially favorable and potentially adverse changes.

Cardiac remodeling may influence intestinal homeostasis through hemodynamic and systemic mechanisms. Altered intestinal perfusion or venous congestion, together with neurohumoral activation, systemic inflammation, and oxidative stress, can affect intestinal tissue integrity and may provide a physiological context for the alterations observed in the CH group [34,35,36,37]. In the present study, sFB administration after hypertrophy induction was associated with changes in intestinal histology and cell-death-related markers. However, intestinal perfusion, epithelial permeability, local inflammation, and oxidative status were not directly evaluated. Therefore, the present findings do not establish restoration of intestinal barrier function or identify the mechanisms underlying the intestinal response associated with sFB.

The hepatic findings are particularly relevant to the assessment of systemic safety. Marked histological alterations were observed under some experimental conditions, particularly when sFB was administered before isoproterenol exposure, and these changes were not consistently accompanied by improvement in the oxidative markers evaluated.

Previous studies have reported different hepatic responses to boron-containing compounds [38,39]. Depending on the compound and experimental conditions, these responses have included changes in hepatic histology and oxidative-status markers, suggesting that chemical form, dose, duration of exposure, and physiological context may influence the hepatic response to boron-containing compounds. However, these findings cannot be directly extrapolated to sFB, and comparisons among different boron compounds should therefore be approached cautiously.

### 3.4. Differential Hepatic Response to sFB Administration

Among the extracardiac tissues evaluated, the liver showed a particularly complex response to sFB administration. In CH-sFB mice, hepatic histological alterations persisted, while GSH and protein carbonyl levels did not differ significantly from those of the untreated CH group. This discordance between histological and biochemical findings does not support interpreting the hepatic response as a defined “intermediate recovery phase,” particularly because only a single post-treatment time point was evaluated. Rather, these findings indicate that changes in antioxidant status may occur without parallel resolution of tissue injury.

The hepatic response was also dependent on the timing of sFB administration. When sFB was administered before isoproterenol exposure (sFB-CH), marked histological alterations were accompanied by reduced GSH levels and elevated circulating TNF-α, indicating that prior sFB administration did not prevent the hepatic alterations observed under this experimental condition. Previous studies have reported both beneficial and adverse hepatic responses to different boron-containing compounds [7,39]. Importantly, experimental evidence indicates that the histopathological effects of boron exposure may vary with both dose and duration of exposure [40]. More broadly, reported biological effects of boron vary with dose, with potentially beneficial effects under some exposure conditions and adverse effects associated with higher exposure levels [41,42]. Although these findings cannot be directly extrapolated to sFB, collectively they suggest that hepatic responses to boron-containing compounds may vary according to chemical form, dose, duration of exposure, and physiological context.

## 4. Materials and Methods

### 4.1. Preparation and Characterization of Synthetic Fructoborate

Synthetic fructoborate (sFB; FB-1) was prepared according to a procedure previously reported by our group [10]. Briefly, D-(−)-fructose (1.00 g, 5.55 mmol) and phenylboronic acid (1.353 g, 11.1 mmol) were suspended in acetone (40 mL), corresponding to a 1:2 molar ratio of fructose to phenylboronic acid. The mixture was stirred under reflux for 180 min, with reaction progress monitored by thin-layer chromatography (TLC). The resulting solution was filtered and crystallized by nucleation under controlled cooling conditions. Crystals formed within 2 days and were collected as a white solid in 74% yield.

The identity of the synthesized FB-1 was assessed by TLC, infrared (IR) spectroscopy, and ^1^H and ^11^B NMR spectroscopy by comparison with previously reported characterization data [10,21]. TLC analysis gave an Rf value of 0.42 using hexane/ethyl acetate (2:3, *v*/*v*) as the mobile phase, and the spectroscopic data were consistent with previously reported values.

^1^H NMR (300 MHz, DMSO-*d_6_*) δ 5.34 (t, *J* = 5.9 Hz, 1H), 5.13 (dd, *J* = 8.4, 2.4 Hz, 1H), 4.79 (m, 2H), 3.83 (dd, *J* = 14, 0.5 Hz, 1H), 3.62 (dd, *J* = 14.1, 1.6 Hz, 1H), 3.34 (m, 2H). ^13^C-NMR (75 MHz, DMSO-*d*6): 136.8, 134.2, 134.1, 134, 131.6, 131.4, 128.4, 127.7, 127.6, 127.4, 124.8, 114.9, 104.7, 71.8, 71.6, 71.1, 63.6.

### 4.2. Evaluation of the Cardiac Effects of Short-Term Synthetic Fructoborate Administration

Twenty-five 6-week-old male BALB/c mice (body weight, 29.2 ± 2.2 g) were obtained from the animal facility of the Hospital Infantil de México Federico Gómez (HIMFG). Animals were housed under controlled lighting conditions with food and water available ad libitum and were maintained in accordance with the Mexican Official Standard NOM-062-ZOO-1999.

In the first experimental phase, the cardiac effects of short-term sFB administration were evaluated using doses selected within the previously reported range of 10–100 mg/kg [10,43]. The 25 mice were randomly allocated to five experimental groups (*n* = 5 per group). Three groups received sFB at doses of 17 mg/kg (sFB-I), 34 mg/kg (sFB-II), or 68 mg/kg (sFB-III) once daily for 3 consecutive days. A fourth group received sodium tetraborate (Bx; 100 mg/kg), which was included as an inorganic boron comparator based on previous work by our group [43], whereas the control group (CTR) received vehicle alone. sFB and Bx were prepared in sterile water for injection and administered by oral gavage using a mouse gastric cannula. The administration volume was adjusted according to body weight. All treatments were administered between 08:00 and 09:00 h.

Sample size was determined using the resource equation method for animal experiments [44], resulting in five animals per group. No animals were excluded from the study, and no deaths occurred during the experimental period. Body weight changes during the 3-day treatment period were not evaluated.

Forty-eight hours after the third administration, mice were anesthetized by intraperitoneal injection of ketamine hydrochloride (100 mg/kg) and xylazine hydrochloride (10 mg/kg). Following laparotomy, tissues were collected for subsequent analyses, and death was confirmed by cervical dislocation. To characterize the cardiac response to short-term sFB administration, cardiac morphology, p38 MAPK phosphorylation, and relative *ANP* and *BNP* mRNA levels were evaluated [45]. Plasma GSH and protein carbonyl levels were also measured as indicators of antioxidant status and oxidative protein modification, respectively.

### 4.3. Cardiac Morphometry

Hearts from mice in each experimental group were perfused with phosphate-buffered saline (PBS) and fixed in 3.5% neutral-buffered formalin. The atria and ventricular inflow and outflow tracts were removed, leaving the midventricular and apical regions. Macroscopic morphometric measurements were performed on the exposed transverse surface of the midventricular region. Each heart was photographed at the same focal distance using a Zeiss stereomicroscope (Carl Zeiss Microscopy, LLC, North Broadway, NY, USA), and images were calibrated before analysis.

Ventricular cross-sectional area was determined by manually tracing the external ventricular contour, whereas left ventricular lumen area was determined by manually tracing the luminal boundary. Left ventricular free-wall thickness was measured at multiple points along the ventricular free wall and averaged to obtain a representative value for each animal. Measurements were performed using ImageJ software version 1.54K (National Institutes of Health, Bethesda, MD, USA) by an investigator blinded to experimental group allocation. One macroscopic image per animal was used for these measurements, and the individual animal was considered the experimental unit for statistical analysis.

### 4.4. Western Blot Analysis

Cardiac p38 MAPK and phosphorylated p38 MAPK (p-p38 MAPK) protein levels were evaluated by Western blotting. β-Actin was used as a loading control. Primary antibodies against p38 MAPK, p-p38 MAPK, and β-actin were used at a dilution of 1:1000. An HRP-conjugated anti-mouse secondary antibody (Thermo Scientific, Waltham, MA, USA) was used at a dilution of 1:15,000. Immunoreactive bands were visualized by chemiluminescence using the Immobilon Western Chemiluminescent HRP Substrate (WBKLS0100; Merck KGaA, Darmstadt, Germany). Band intensities were quantified by densitometric analysis using ImageJ software (National Institutes of Health, Bethesda, MD, USA). The relative phosphorylation of p38 MAPK was expressed as the p-p38 MAPK/p38 MAPK ratio.

### 4.5. sFB Administration in the Isoproterenol-Induced Cardiac Hypertrophy Model

**Figure 10:**
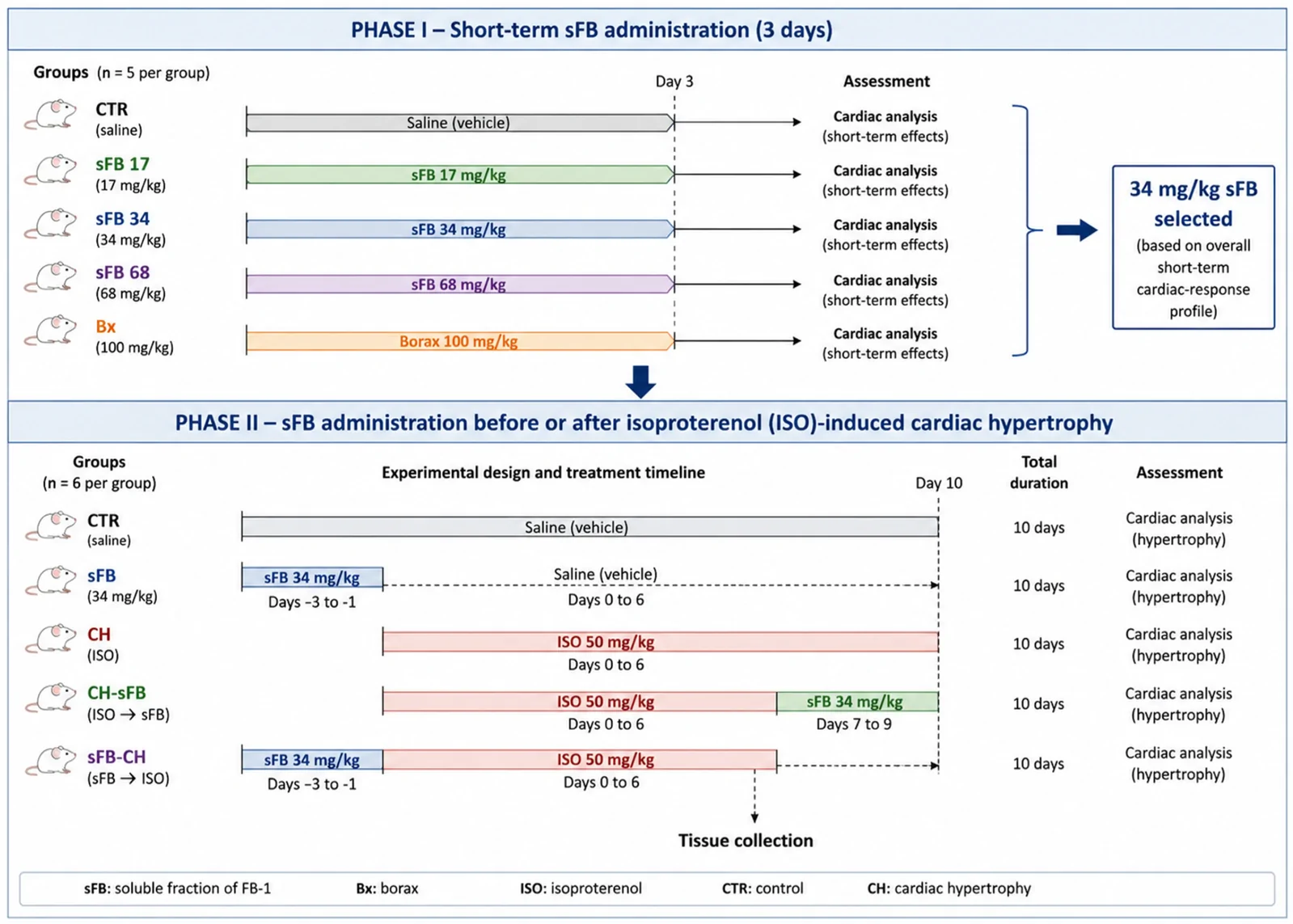
Experimental design and timeline of the study. Phase I evaluated the cardiac effects of short-term sFB administration and supported the selection of 34 mg/kg for Phase II, which compared sFB administration before and after isoproterenol-induced cardiac hypertrophy. sFB was administered intragastrically at 34 mg/kg for 3 consecutive days. ISO was administered intraperitoneally at 50 mg/kg for 7 consecutive days. CTR, control; sFB, synthetic fructoborate; CH, cardiac hypertrophy; ISO, isoproterenol. *n* = 6 mice per group. The horizontal arrow indicates the selected FB dose for the second phase. The down arrow describes the protocol for the second phase.

In the second phase of the study, the effects of sFB administration before or after induction of cardiac hypertrophy were evaluated. Thirty male BALB/c mice (body weight, 25.8 ± 1.2 g) were randomly allocated to five experimental groups (*n* = 6 per group), as described in Table 1 [44]. Figure 10 illustrates the overall experimental design and treatment timeline. Based on the findings from the short-term administration experiment, an sFB dose of 34 mg/kg was selected for this phase. Cardiac hypertrophy was induced by intraperitoneal administration of isoproterenol (ISO; 50 mg/kg) for 7 consecutive days [46,47]. Control mice (CTR) received saline instead of ISO. No deaths occurred in any experimental group.

Depending on the experimental group, sFB (34 mg/kg) was administered intragastrically for 3 consecutive days either before or after induction of cardiac hypertrophy (Figure 10). At the end of the experimental period, mice were anesthetized with xylazine (10 mg/kg; Xylazine Injectable Aranda, Aranda, Querétaro, México) and Zoletil^®^ 100 (20 mg/kg; Vibarc SA, Guadalajara, México). The heart, lung, intestine, kidney, and liver were collected. Tissue samples intended for histological analysis were fixed in neutral-buffered formalin, whereas samples intended for molecular analyses were immediately frozen in liquid nitrogen and stored until use.

### 4.6. Tissue Histology

Formalin-fixed heart, lung, liver, and ileal tissues were fixed in 3.5% neutral-buffered formalin for 24–72 h, dehydrated through a graded ethanol series, cleared, and embedded in paraffin. Tissue sections (4–5 µm thick) were obtained, mounted on glass slides, deparaffinized, rehydrated, and stained with hematoxylin and eosin (H&E) for histological evaluation.

For cardiac histological assessment, five serial sections per animal were examined at the level of the left ventricular free wall. Five randomly selected fields per section were acquired at 40× magnification using a conventional bright-field microscope. Interstitial space was quantified using ImageJ software after conversion of the images to binary format. Measurements from the analyzed fields and sections were averaged to obtain a single value for each animal, with the individual animal considered the experimental unit.

### 4.7. Wheat Germ Agglutinin Staining

Heart sections were stained with wheat germ agglutinin conjugated to Alexa Fluor™ 488 (WGA–Alexa Fluor™ 488; Thermo Scientific, Waltham, MA, USA) to visualize cardiomyocyte boundaries. Five sections were analyzed, and five micrographs were acquired per section at 40× magnification with 1.5× digital zoom, for a total of 25 micrographs. Only cardiomyocytes observed in transverse section and with clearly defined WGA-stained boundaries were included. Cardiomyocyte contours were manually traced using ZEN 2009 software. The control-group value was set to 100%, and values from the remaining groups were expressed relative to the control.

### 4.8. Immunofluorescence

Paraffin-embedded heart, lung, and ileal tissue sections were deparaffinized and rehydrated. Following antigen retrieval, sections were washed with PBS containing Tween 20 (PBS-T) and incubated overnight at 4 °C with the corresponding primary antibodies: anti-collagen I and anti-collagen III for heart sections, anti-MMP-13 for lung sections, and anti-caspase-8 for ileal sections.

After washing with PBS-T, the corresponding Alexa Fluor™ 488- or Alexa Fluor™ 594-conjugated anti-mouse or anti-rabbit secondary antibodies were applied for 4 h at room temperature. Nuclei were counterstained with DRAQ7, and sections were mounted using PBS/glycerol (1:1, *v*/*v*).

Samples were examined using a Zeiss LSM 510 confocal microscope with a 40× objective and 2× digital zoom. For each marker, images used for quantitative comparisons among experimental groups were acquired using identical confocal acquisition settings. Fluorescence intensity was quantified using ZEN 2009 software. Measurements obtained from multiple fields/sections from the same animal were averaged to obtain a single value per animal, and the individual animal was considered the experimental unit.

### 4.9. TUNEL Assay

DNA fragmentation in tissue sections was evaluated using the terminal deoxynucleotidyl transferase dUTP nick-end labeling (TUNEL) assay. Paraffin-embedded tissue sections were deparaffinized and rehydrated through a graded ethanol series. TUNEL staining was performed using the In Situ Cell Death Detection Kit (Cat. No. 11684795910; Roche Diagnostics, Basel, Switzerland) according to the manufacturer’s instructions. Nuclei were counterstained with DRAQ7, and sections were mounted using PBS/glycerol as the mounting medium. Samples were examined using a Zeiss inverted confocal microscope (Oberkochen, Germany), and images were acquired using ZEN 2009 software.

### 4.10. Quantification of Protein Carbonyls

Protein carbonyl levels were determined using a modified 2,4-dinitrophenylhydrazine (DNPH) assay [48,49,50]. Briefly, an equal volume of DNPH solution was added to each plasma sample, and the mixture was incubated for 10 min in the dark with continuous shaking. Subsequently, 0.6 M NaOH was added, and the samples were incubated with shaking for an additional 10 min. Protein carbonyl content was then determined spectrophotometrically by measuring absorbance at 450 nm.

### 4.11. Determination of GSH Levels

Plasma samples were mixed with an equal volume of 10% (*v*/*v*) metaphosphoric acid and centrifuged at 3000× *g* for 10 min at 4 °C. The resulting supernatants were collected, and reduced glutathione (GSH) levels were determined spectrophotometrically using Ellman’s reagent [5,5′-dithiobis (2-nitrobenzoic acid), DTNB], as previously described [51]. Absorbance was measured at 412 nm and used to determine GSH concentration.

### 4.12. Plasma TNF-α Measurement

Plasma TNF-α levels were measured using a MILLIPLEX^®^ MAP multiplex immunoassay according to the manufacturer’s instructions. Samples were analyzed using a MAGPIX^®^ system, and TNF-α concentrations were determined from the corresponding standard curve.

### 4.13. Reverse Transcription Quantitative PCR (RT-qPCR)

Total RNA was extracted using TRIzol^®^ Reagent (Invitrogen) according to the manufacturer’s instructions. Complementary DNA (cDNA) was synthesized from 1 µg of total RNA in a final reaction volume of 20 µL using M-MuLV reverse transcriptase (New England Biolabs, Ipswich, MA, USA), according to the manufacturer’s instructions. Gene-specific primers were used to determine the relative mRNA levels of *ANP*, *BNP*, *ADRB1*, *c-jun*, *c-fos*, *c-myc*, *Gata-4*, *Nkx2.5*, *Bax*, *Bcl-2*, and *p53* [47,48]. Primer sequences are provided in Table 1. Quantitative PCR was performed using SYBR Green chemistry on an AriaMx Real-Time PCR System (Agilent Technologies, Santa Clara, CA, USA), and data were acquired using Agilent Aria software v1.3. Each biological sample was analyzed in technical triplicate in a final reaction volume of 20 µL containing 1× SYBR Green Master Mix (Thermo Fisher Scientific, Waltham, MA, USA), 0.4 µM each of forward and reverse primers, nuclease-free water, and 100 ng of cDNA. Amplification conditions consisted of an initial denaturation at 95 °C for 10 min, followed by 40 cycles at 95 °C for 15 s and 56 °C for 60 s. GAPDH was used as the reference gene. Relative mRNA expression was calculated using the [2^−ΔΔCt^] method. Table 1 shows the primer sequences used in this study.

### 4.14. Statistical Analysis

Data were analyzed using parametric statistical methods. Normality and homogeneity of variance were assessed using the Shapiro–Wilk and Levene tests, respectively. Differences among experimental groups were evaluated using one-way analysis of variance (ANOVA). When a significant overall effect was detected, Tukey’s multiple-comparisons test was used for pairwise comparisons among groups. Data are presented as the mean ± standard deviation (SD). A *p* value < 0.05 was considered statistically significant. Statistical analyses were performed using SPSS Statistics version 22.0 (IBM, Armonk, NY, USA).

## 5. Conclusions

Under the experimental conditions evaluated, short-term administration of sFB did not produce a consistent pattern of structural or molecular changes indicative of overt cardiac injury. In the isoproterenol-induced cardiac hypertrophy model, sFB administration after hypertrophy induction was associated with attenuation of several features of cardiac structural remodeling, including left ventricular wall thickness, cardiomyocyte area, and collagen deposition. In contrast, administration of sFB before isoproterenol exposure produced a different response and did not uniformly prevent the structural and molecular changes associated with cardiac hypertrophy.

Extracardiac responses were heterogeneous and varied across tissues and according to the timing of sFB administration, with hepatic findings highlighting the need for further safety evaluation. Overall, these results indicate that the biological responses associated with sFB in this model varied according to the experimental context and timing of administration. Longer-term studies incorporating cardiac and organ-specific functional assessments, pharmacokinetics, tissue distribution, and additional molecular analyses are needed to better define the safety profile and potential therapeutic relevance of sFB.

## 6. Limitations

Several limitations should be considered when interpreting the present findings. First, although the isoproterenol-induced cardiac hypertrophy model is widely used to study cardiac remodeling, it does not reproduce the full complexity of human disease. In addition, only male mice were included; therefore, possible sex-dependent responses to sFB remain unknown.

The short duration of sFB administration is another important limitation. The 3-day treatment was intended as an initial assessment of the biological response to sFB and should not be interpreted as demonstrating cardiovascular or systemic safety. The limited number of animals and the evaluation of selected experimental endpoints also limit conclusions regarding long-term effects and the temporal progression of the responses observed.

Several extracardiac effects were evaluated using histological observations and selected molecular and biochemical markers. Changes in p53 mRNA expression or the Bax/Bcl-2 mRNA ratio, for example, cannot by themselves establish activation or inhibition of apoptosis. Similarly, these markers cannot be considered direct measures of organ function or toxicity. In addition, the signaling pathways discussed as potential contributors to the observed responses, including TGF-β/Smad, calcineurin/NFAT, and intracellular Ca^2+^ handling, were not directly evaluated and should therefore be considered hypotheses for future investigation.

Finally, the study focused primarily on morphological, histological, molecular, and biochemical endpoints and did not include echocardiographic assessment of cardiac function or gross cardiac hypertrophy indices, such as heart weight/body weight and heart weight/tibia length ratios. Therefore, the structural changes observed in this study cannot be assumed to reflect improvements in cardiac function. Pharmacokinetics, tissue distribution, and metabolism of sFB were also not evaluated. Future studies incorporating functional cardiac assessment, gross hypertrophy indices, longer treatment periods, and pharmacokinetic analyses will be necessary to better define the biological significance and potential therapeutic relevance of sFB.

## Figures and Tables

**Table 1:** Primer sequences used in this study.

Primer	Forward	Reverse
*ANP*	TCC GAT AGA TCT GCC CTC TT	CTC CAA TCC TGT CAA TCC TAC C
*BNP*	ACT CCT ATC CTC TGG GAA GTC	GCT GTC TCT GGG CCA TTT
*ADRB1*	AGT TCT GGC TGA GAG GGA CA	GGG GGT GTG GAG GAG ATA AT
*c-jun*	AAA ACC TTG AAA GCG CAA AA	CGC AAC CAG TCA AGT TCT CA
*c-fos*	CTC CCG TGG TCA CCT GTA CT	TTG CCT TCT CTG ACT GCT CA
*c-myc*	GCC CAG TGA GGA TAT CTG GA	ATC GCA GAT GAA GCT CTG GT
*Gata-4*	GCA GCA GCA GTG AAG AGA TG	GCG ATG TCT GAG TGA CAG GA
*Nkx2.5*	CCA CTT AGG CAT TTC CCA GA	GGG AAG AGA GGC AGA GAG GT
*Bax*	TCC AGG ATC GAG CAG A	AAG TAG AAG AGG GCA ACC
*Bcl-2*	GGA GGA TTG TGG CCT TCT TT	GTC ATC CAC AGA GCG ATG TT
*p53*	ACA TGA CTG AGG TCG TGA GA	GAT TTC CTT CCA CCC GGA TAA G
*GAPDH*	TTC CAT CCT CCA GAA ACC AG	CCC TCG AAC TAA GGG GAA AG

## Data Availability

The data supporting the findings of this study are included in the article. Additional information is available from the corresponding author upon reasonable request.

## Abbreviations

The following abbreviations are used in this manuscript:

*ADRB1*	β1-adrenergic receptor
*ANP*	Atrial natriuretic peptide
*Bax*	Bcl-2-associated X protein
Bcl-2	B-cell lymphoma 2
*BNP*	B-type natriuretic peptide
*CASP8*	Caspase-8
c-*Jun*	Jun proto-oncogene
CTR	Control group
*c-Fos*	Fos proto-oncogene
c-*Myc*	MYC proto-oncogene
CH	Cardiac hypertrophy
DNPH	2,4-Dinitrophenylhydrazine
DTNB	5,5′-Dithiobis(2-nitrobenzoic acid)
*Gata4*	Gata-binding protein 4
GAPDH	Glyceraldehyde-3-phosphate dehydrogenase
GPCR	G protein-coupled receptor
GSH	Glutathione
sFB	Synthetic fructoborate
H&E	Hematoxylin and eosin
IPA	Ingenuity Pathway Analysis
LV	Left ventricle
LVH	Left ventricular hypertrophy
LVWT	Left ventricular wall thickness
MMP-13	Matrix metalloproteinase-13
mRNA	Messenger RNA
NaOH	Sodium hydroxide
*Nkx2.5*	NK2 homeobox 5
p38 MAPK	p38 mitogen-activated protein kinase
p53	Tumor protein p53
TNF-α	Tumor necrosis factor alpha
TUNEL	Terminal deoxynucleotidyl transferase dUTP nick-end labeling
qPCR	Quantitative polymerase chain reaction
WGA	Wheat germ agglutinin
